# Prognostic value of T lymphocyte CD40L expression levels in patients with sepsis

**DOI:** 10.3389/fmed.2026.1949041

**Published:** 2026-10-01

**Authors:** Bin Shao, Ziyi Huang, Yujie Liu, Zhiyang Wang, Fang Huang, Jun Wang

**Affiliations:** 1 Department of Critical Care Medicine, The First Affiliated Hospital of Soochow University, Suzhou, China; 2 Department of Critical Care Medicine, Qingdao Municipal Hospital, Qingdao, China; 3 Jiangsu Institute of Clinical Immunology, The First Affiliated Hospital of Soochow University, Suzhou, China; 4 Department of Emergency and Critical Care Medicine, The Second Affiliated Hospital of Soochow University, Suzhou, China

**Keywords:** 28-day mortality, CD4+ and CD8+ T lymphocytes, CD40L, prognostic biomarker, sepsis

## Abstract

**Background:**

This study aimed to analyze the expression characteristics of CD40L on peripheral blood T lymphocytes in patients with sepsis and explore its value in assessing disease severity and prognosis.

**Methods:**

This prospective study collected blood samples and clinical data from adult sepsis patients admitted to the Department of Critical Care Medicine at the First Affiliated Hospital of Soochow University between March 2023 and March 2024, with the primary endpoint being 28-day mortality. Multivariate Cox regression analysis was performed to evaluate the association between T lymphocyte CD40L expression and 28-day mortality. Additionally, machine learning algorithms were applied to determine the importance of CD40L-related features, and latent class mixed model analysis was conducted to identify different expression trajectories.

**Results:**

A total of 112 sepsis patients were included, with a 28-day mortality rate of 20.5%. CD40L expression levels on peripheral blood CD4^+^ and CD8^+^ T lymphocytes were significantly higher in sepsis patients compared to healthy controls. Three distinct expression patterns were identified for both CD4^+^ and CD8^+^ T cells. Patients with persistently low CD40L expression levels had significantly lower survival rates (*P* < 0.001). Moreover, multivariate Cox analysis identified day 1 CD40L levels on CD4^+^ (HR 0.712, 95% CI 0.606–0.837, *P* < 0.001) and CD8^+^ T cells (HR 0.593, 95% CI 0.481–0.731, *P* < 0.001) as an independent predictor of 28-day mortality. ROC analysis revealed strong associations between day 1 CD40L expression and mortality, with AUCs of 0.847 and 0.875, respectively. Finally, the combination of CD40L expression with SOFA scores improved clinical decision-making compared to SOFA scoring alone.

**Conclusion:**

Peripheral blood T lymphocyte CD40L was identified as an independent predictor of 28-day mortality in sepsis patients. Identifying distinct expression trajectories of CD40L in peripheral blood T lymphocytes can effectively assist in predicting short-term prognosis in patients with sepsis.

## Introduction

As is well documented, sepsis is a life-threatening syndrome caused by a dysregulated host response to infection, resulting in organ dysfunction (1). It is not only one of the leading causes of death among critically ill patients but also a pressing global public health challenge. According to statistics, approximately 49 million cases of sepsis are recorded worldwide each year, with over 11 million related deaths, accounting for 19.7% of global mortality. Thus, sepsis has emerged as a major disease threatening human health (2).

Currently, the treatment of sepsis is predominantly nonspecific and largely supportive in nature, aiming to reduce mortality through early fluid resuscitation and timely administration of broad-spectrum antibiotics. However, there is significant heterogeneity among patients in terms of the causative pathogens, sex, age, comorbidities, and genetic background, all of which lead to varying immune responses. Despite this, clinical treatment strategies are generally standardized. As a result, while a given therapeutic approach may benefit one patient, it could be harmful to another due to differences in their immune responses to infection. This discrepancy can lead to significantly different outcomes in patients who present with seemingly similar symptoms and clinical manifestations (3).

Therefore, the ability to accurately and rapidly identify sepsis and its associated immune states is crucial for informing public health interventions and concurrently developing individualized clinical treatment strategies.

During the onset and progression of sepsis, immunosuppression, functional impairment of immune cells, and apoptosis of lymphocytes are commonly observed, accompanied by significant alterations in the levels of various biomarkers and cytokines. Flow cytometry has been adopted in clinical practice, enabling detailed immunophenotyping of immune cell subsets. This technique provides comprehensive analytical reports and allows for the dynamic monitoring of both quantitative changes in lymphocyte subsets and the expression profiles of surface markers (4–6). Consequently, it offers valuable insights for assessing immune status and evaluating disease progression. For instance, flow cytometric measurement of leukocyte biomarkers has demonstrated that clinical deterioration in sepsis is associated with a combination of neutrophil CD24 and CD279 (PD-1) expression, as well as HLA-DR expression levels on monocytes (7).

CD40 is a costimulatory receptor molecule and a member of the tumor necrosis factor receptor superfamily, specifically TNFRSF5. Its ligand, CD40L (CD154), is primarily expressed on CD4⁺ T cells and endothelial cells, with lower expression levels on activated platelets and various immune cells, including CD8⁺ T cells, B cells, dendritic cells, and macrophages. The CD40–CD40L interaction provides an essential costimulatory signal for the activation of both T lymphocytes and B lymphocytes (8, 9). As a key molecule in immune regulation, CD40L participates in the modulation of adaptive immune responses through a dual mechanism of action. In T cell–mediated immune responses, CD40L binds specifically to CD40 receptors on the surface of antigen-presenting cells (APCs), initiating a costimulatory signaling cascade. This interaction is essential for the full activation of T lymphocytes and the execution of their effector functions (10). In B–cell–mediated humoral immune responses, CD40L delivers a secondary activation signal via the CD40–CD40L axis, which not only promotes the proliferation and differentiation of B lymphocytes but also regulates key molecular processes such as **class-switch recombination (CSR)** and **somatic hypermutation (SHM)**. Through these mechanisms, the CD40–CD40L interaction enhances both the quality and quantity of the antibody response (11).

In summary, monitoring the quantity of lymphocyte subsets can more accurately reflect a patient's immune status and disease severity, thereby offering novel therapeutic insights for clinical management. This study aimed to investigate the relationship between the expression levels and dynamic changes of CD40L on CD4⁺ and CD8⁺ T lymphocytes in patients with sepsis, as well as their association with disease severity and prognosis. The findings are expected to provide a scientific reference for the identification of potential immunotherapeutic targets in the treatment of sepsis.

## Methods

### Study population

This prospective observational study consecutively enrolled adult patients who met the diagnostic criteria for Sepsis 3.0 and were treated at the Department of Critical Care Medicine at the First Affiliated Hospital of Soochow University. Additionally, 20 healthy adults were recruited as a control group. The study was conducted in accordance with the principles of medical ethics and was approved by the Ethics Committee of the First Affiliated Hospital of Soochow University. Informed consent was obtained from all patients and/or their legal guardians. Diagnostic criteria for sepsis were as follows: (1) A confirmed or suspected infection; (2) An increase in the Sequential Organ Failure Assessment (SOFA) score of ≥ 2 points from baseline.

Inclusion criteria were as follows: (1) Age ≥ 18 years; (2) Meeting the diagnostic criteria for Sepsis 3.0; (3) For patients with multiple ICU admissions, only data from the first ICU admission were included. Exclusion criteria were as follows: (1) Pregnancy or lactation; (2) Presence of malignancies or active cancer; (3) Hematologic malignancies; (4) History of organ transplantation; (5) Rheumatic or autoimmune diseases; (6) Recent receipt of immunotherapy; (7) Time from onset to admission exceeding 48 h. Withdrawal criteria encompassed: (1) ICU length of stay less than 24 h; (2) Incomplete data or loss to follow-up; (3) Withdrawal from the study for any reason (Figure 1).

**Figure 1:**
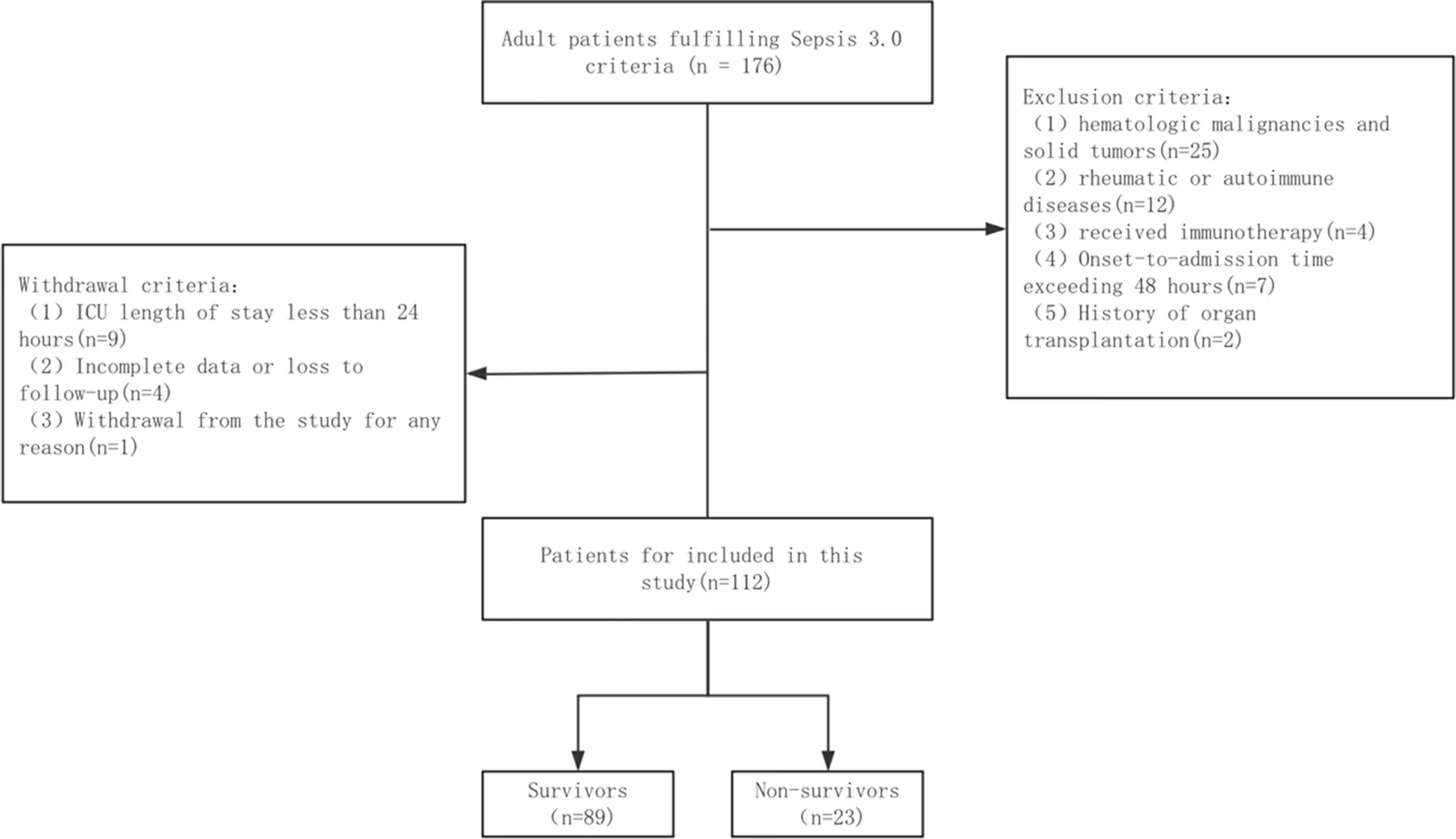
Inclusion/exclusion criteria.

### Procedures

Peripheral blood samples were collected from sepsis patients on days 1, 3, and 7 following ICU admission. Flow cytometry was performed to monitor lymphocyte subsets, specifically measuring the proportion of CD4⁺ T cells and CD8⁺ T cells expressing CD40L. Baseline clinical and laboratory data were also collected, including: white blood cell count (WBC), lymphocyte count (LY), hemoglobin (HB) level, platelet count (PLT), total bilirubin level (TBIL), alanine aminotransferase (ALT) level, aspartate aminotransferase (AST) level, creatinine level (UREA), albumin (ALB) level, prothrombin time (PT), activated partial thromboplastin time (APTT), C-reactive protein level (CRP), procalcitonin level (PCT), interleukin-6 level (IL-6), interleukin-8 (IL-8) level, interleukin-10 (IL-10) level, pH level, lactate (Lac) level, and oxygenation index (PaO₂/FiO₂). The primary outcome of this study was 28-day mortality following ICU admission.

### Statistical analysis

Statistical analyses and figure generation were performed using R software (version 4.4.2). For categorical variables, Fisher's exact test or the Chi-square test was used for between-group comparisons, with results presented as counts and percentages. For continuous variables, the Wilcoxon rank-sum test or one-way analysis of variance (ANOVA) was applied. Continuous variables with a normal distribution were expressed as mean ± standard deviation (SD), whereas non-normally distributed variables were reported as median and interquartile range (IQR). *P*-value < 0.05 was considered statistically significant.

A latent class mixed model (LCMM) was employed to explore the dynamic expression patterns of CD40L, identifying distinct expression trajectories. Kaplan–Meier survival curves were plotted to compare 28-day survival among expression groups, and the log-rank test was used to evaluate statistical differences. Spearman's correlation analysis was conducted to assess relationships between variables. The Boruta algorithm was utilized to select features associated with 28-day prognosis, and SHAP (SHapley Additive exPlanations) analysis was applied to evaluate the relative contribution of selected variables.

Significant predictors were further incorporated in a multivariate Cox proportional hazards regression model to identify independent risk factors for 28-day mortality in sepsis patients. To assess the predictive performance of each indicator for 28-day mortality, receiver operating characteristic (ROC) curves were generated, and decision curve analysis (DCA) was performed to evaluate clinical utility.

## Results

### Baseline characteristics

A total of 112 patients with sepsis were included in this study. In the survival group, the median age was 70.00 years [IQR: 53.00–78.00], with 32 females (36%) and a median BMI of 23.44 [20.83–26.17] (Table 1). In the non-survival group, the median age was 74.00 years [63.50–80.50], with 6 females (26.1%) and a median BMI of 22.49 [21.24–25.67]. Age, sex, and BMI were comparable between the two groups. Regarding comorbidities, the most prevalent condition in the overall cohort was hypertension, present in 67 patients (59.8%), followed by diabetes mellitus in 34 patients (30.4%). The prevalence of diabetes was significantly higher in the survival group compared to the non-survival group (36% vs. 8.7%, *P* = 0.023), while no significant differences were observed between the groups for other comorbidities. Analysis of infection sites revealed that respiratory tract infections were the leading cause of hospitalization among patients with sepsis, accounting for 46.4% of cases, followed by gastrointestinal infections at 25%. No statistically significant differences in infection sites were detected between the survival and non-survival groups. Concerning critical illness severity scores, the survival group had significantly lower APACHE II scores (15.00 [11.00–19.00] vs. 22.00 [15.00–24.00], *P* = 0.001) and SOFA scores (6.00 [4.00–8.00] vs. 10.00 [7.50–13.50], *P* < 0.001) compared to the non-survival group. The proportion of patients requiring mechanical ventilation [30 (33.7%) vs. 19 (82.6%), *P* < 0.001], vasoactive agents [40 (44.9%) vs. 20 (87.0%), *P* = 0.001], and continuous renal replacement therapy (CRRT) [21 (23.6%) vs. 14 (60.9%), *P* = 0.001] was significantly lower in the survival group compared to the non-survival group. In contrast, no significant differences were noted between the two groups in terms of ICU length of stay or total hospital length of stay. Lastly, the levels of lactate, IL-6, IL-10, total bilirubin, ALT, AST, PT, APTT, and high-sensitivity troponin were significantly lower in the survival group compared to the non-survival group.

**Table 1:** Baseline characteristics of the survival and non-survival groups.

Characteristic	Overall (*n* = 112)	Survival (*n* = 89)	Non-survival (*n* = 23)	*P*
Demographic				
Age	71.5 [53.75, 78.25]	70.0 [53.00, 78.00]	74.00 [63.50, 80.50]	0.296
Gender (male)	38 (33.9)	32 (36.0)	6 (26.1)	0.520
BMI	23.18 [20.83, 25.99]	23.44 [20.83, 26.17]	22.49 [21.24, 25.67]	0.503
Comorbidities				
Hypertension	67 (59.8)	54 (60.7)	13 (56.5)	0.902
Diabetes	34 (30.4)	32 (36.0)	2 (8.7)	0.023
COPD	6 (5.4)	4 (4.5)	2 (8.7)	0.781
Stroke	16 (14.3)	13 (14.6)	3 (13.0)	1.000
CAD	8 (7.1)	5 (5.6)	3 (13.0)	0.436
HF	6 (5.4)	3 (3.4)	3 (13.0)	0.188
CKD	9 (8.0)	9 (10.1)	0 (0.0)	0.246
Infection				
Pulmonary	52 (46.4)	37 (41.6)	15 (65.2)	0.073
Urinary	15 (13.4)	14 (15.7)	1 (4.3)	0.278
Blood	13 (11.6)	11 (12.4)	2 (8.7)	0.901
Abdominal	28 (25.0)	25 (28.1)	3 (13.0)	0.224
Other	4 (3.6)	2 (1.1)	2 (8.3)	0.392
APACHE II	16.00 [12.00, 21.00]	15.00 [11.00, 19.00]	22.00 [15.00, 24.00]	0.001
SOFA	7.00 [5.00, 10.00]	6.00 [4.00, 8.00]	10.00 [7.50, 13.50]	<0.001
Events				
MV	49 (43.8)	30 (33.7)	19 (82.6)	<0.001
Vasopressor	60 (53.6)	40 (44.9)	20 (87.0)	0.001
CRRT	35 (31.2)	21 (23.6)	14 (60.9)	0.001
ICU day	11.00 [7.00, 20.00]	11.00 [8.00, 20.00]	10.00 [5.00, 20.50]	0.599
Hospital day	14.00 [9.00, 24.25]	14.00 [9.00, 24.00]	14.00 [5.50, 24.50]	0.364
Laboratory tests				
pH	7.41 [7.35, 7.45]	7.41 [7.35, 7.45]	7.38 [7.34, 7.46]	0.724
Lactate (mmol/L)	1.60 [1.10, 2.50]	1.40 [1.10, 2.08]	2.80 [1.60, 4.25]	0.001
WBC (K/µL)	13.12 [8.40, 19.12]	13.29 [9.12, 19.86]	11.26 [7.24, 15.76]	0.160
Lymphocyte (K/µL)	0.59 [0.39, 0.89]	0.63 [0.42, 0.96]	0.52 [0.36, 0.76]	0.141
Hemoglobin (g/L)	108.00 [96.00, 121.00]	108.00 [93.00, 120.50]	106.00 [96.50, 121.50]	0.850
Platelet (K/µL)	147.00 [90.00, 225.00]	149.00 [105.00, 231.25]	116.00 [51.50, 188.00]	0.09
Procalcitonin (ng/mL)	14.00 [2.03, 52.00]	14.00 [2.07, 52.00]	13.73 [1.90, 43.06]	0.622
hs-CRP (mg/L)	172.98 [89.47, 263.40]	186.79 [108.95, 268.87]	116.13 [57.52, 212.47]	0.05
IL-1 (pg/mL)	3.72 [1.27, 15.81]	3.50 [1.19, 15.55]	5.54 [1.52, 16.37]	0.713
IL-6 (pg/mL)	66.02 [18.94, 167.15]	55.07 [13.55, 165.10]	86.80 [53.23, 281.20]	0.038
IL-8 (pg/mL)	28.80 [4.39, 89.90]	28.58 [2.17, 85.08]	53.16 [5.02, 146.61]	0.189
IL-10 (pg/mL)	3.42 [1.57, 8.37]	2.63 [0.71, 7.68]	4.82 [3.22, 12.20]	0.027
T-Bil (*μ*mol/L)	15.30 [10.20, 27.07]	13.70 [9.20, 24.30]	22.10 [16.45, 50.85]	0.005
ALT (U/L)	30.00 [15.00, 60.00]	28.80 [13.87, 58.10]	41.30 [19.40, 119.85]	0.055
AST (U/L)	39.60 [18.80, 78.00]	30.80 [17.77, 60.88]	61.50 [37.30, 129.50]	0.003
Albumin (g/L)	31.40 [28.90, 34.10]	31.30 [28.70, 34.20]	32.30 [29.55, 33.35]	0.594
Creatinine (μmol/L)	98.00 [70.00, 167.60]	93.75 [67.00, 158.60]	112.00 [85.80, 182.25]	0.327
Glucose (mmol/L)	9.41 [6.96, 12.75]	9.81 [7.23, 13.08]	7.98 [6.48, 10.22]	0.113
Potassium (mEq/L)	3.87 [3.56, 4.23]	3.85 [3.56, 4.17]	4.09 [3.56, 4.72]	0.17
Sodium (mEq/L)	140.40 [137.05, 143.20]	140.05 [136.90, 142.85]	140.70 [137.40, 144.55]	0.426
PT	16.00 [14.85, 17.60]	15.65 [14.67, 16.80]	17.60 [15.80, 21.30]	<0.001
APTT	43.60 [38.20, 51.35]	42.75 [37.40, 50.55]	46.50 [40.75, 54.00]	0.025
NT-proBNP	4330 [1390.00, 13945.00]	4330.00 [1212.00, 13830.00]	3903.00 [1759.75, 14179.00]	0.640

Continuous variables following a normal distribution are expressed as mean ± standard deviation (SD), whereas continuous variables not following a normal distribution are presented as median and interquartile range (IQR). Categorical variables are presented as frequency and percentage [n(%)].

BMI, body mass index; WBC, white blood cell; COPD, chronic obstructive pulmonary disease; CAD, coronary artery disease; HF, heart failure; CKD, chronic kidney disease; SOFA, sequential organ failure assessment; APACHE II, acute physiology and chronic health evaluation II; MV, mechanical ventilation; CRRT, continuous renal replacement therapy; T-Bil, total bilirubin; hs-CRP, high-sensitivity C-reactive protein; PT, prothrombin time; APTT, activated partial thromboplastin time; ALT, alanine aminotransferase; AST, aspartate aminotransferase; NT-proBNP, N-terminal pro b-type natriuretic peptide.

Furthermore, this study quantitatively analyzed the expression of surface CD40L molecules on peripheral blood T lymphocytes from 20 healthy volunteers using flow cytometry and compared the obtained data with corresponding indicators from 112 sepsis patients. The results uncovered that CD40L expression on the surface of CD4^+^ and CD8^+^ T lymphocytes in healthy individuals was low or absent (Figure 2). The expression levels of CD40L on CD4^+^ and CD8^+^ T lymphocytes in sepsis patients on days 1, 3, and 7 were significantly higher than those in healthy individuals.

**Figure 2:**
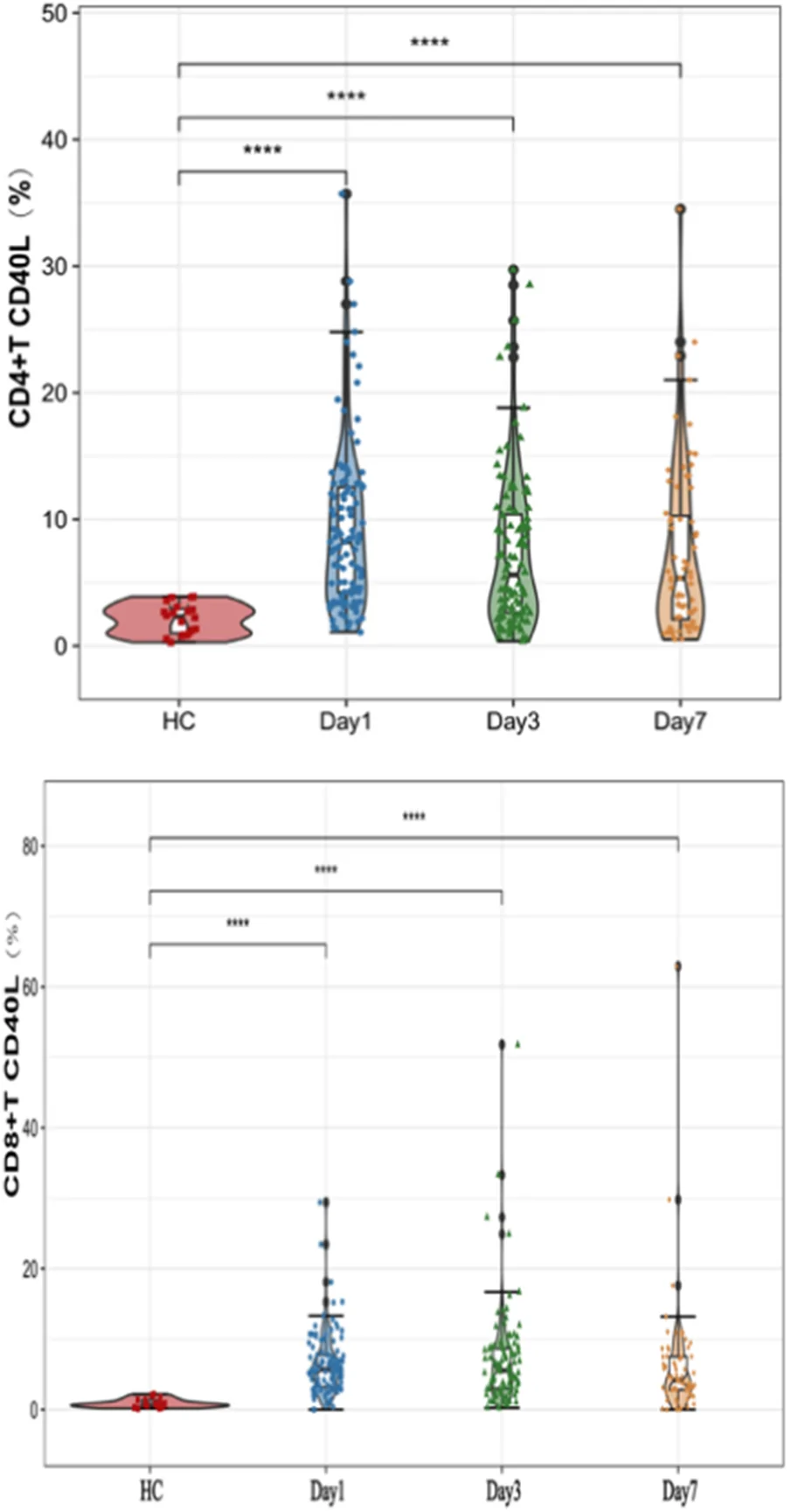
CD40L expression in the T lymphocytes of sepsis patients and healthy controls. Comparisons versus healthy controls were performed using Dunnett's test. *P < 0.05, ** *P* < 0.01, *** *P* < 0.001, **** *P* < 0.0001, ns *P* > 0.05, no statistically significant difference.

CD4 + and CD8+ T cell subsets expressing CD40L were identified using flow cytometry. The analysis showed that the expression levels of CD40L on CD4+ T lymphocytes were significantly higher in the survival group compared to the non-survival group on Day 1 (9.70% [5.45, 12.90] vs. 3.29% [2.22, 6.35], *P* < 0.001), Day 3 (7.54% [3.06, 11.14] vs. 2.73% [1.56, 4.46], *P* = 0.001), and Day 7 (5.98% [3.16, 12.52] vs. 1.40% [0.95, 1.67], *P* < 0.001). Similarly, the expression levels of CD40L on CD8+ T lymphocytes in the survival group were higher than those in the non-survival group on Day 1 (6.62% [5.02, 8.30] vs. 2.50% [1.64, 2.90], *P* < 0.001), Day 3 (6.33% [3.82, 8.92] vs. 2.65% [1.72, 3.79], *P* < 0.001), and Day 7 (4.86% [3.10, 8.59] vs. 0.77% [0.10, 2.16], *P* < 0.001) (Table 2).

**Table 2:** CD40L expression on T lymphocytes.

Characteristic	Overall (*n* = 112)	Survival (*n* = 89)	Non-survival (*n* = 23)	*P*
CD4 ^+^ TC CD40L (%)				
Day 1	8.22 [4.24, 12.52]	9.70 [5.45, 12.90]	3.29 [2.22, 6.35]	<0.001
Day 3	5.61 [2.65, 10.38]	7.54 [3.06, 11.14]	2.73 [1.56, 4.46]	0.001
Day 7	5.34 [2.08, 10.27]	5.98 [3.16, 12.52]	1.40 [0.95, 1.67]	<0.001
CD8 ^+^ TC CD40L (%)				
Day 1	5.70 [3.23, 7.72]	6.62 [5.02, 8.30]	2.50 [1.64, 2.90]	<0.001
Day 3	5.54 [2.97, 8.66]	6.33 [3.82, 8.92]	2.65 [1.72, 3.79]	<0.001
Day 7	4.18 [2.80, 7.52]	4.86 [3.10, 8.59]	0.77 [0.10, 2.16]	<0.001

Median [interquartile range] are shown. *P*-values were Bonferroni-adjusted for six pairwise comparisons between survivors and non-survivors.

Moreover, dynamic changes in the expression levels of CD40L on T lymphocytes in septic patients were assessed on days 1, 3, and 7 using Latent Class Mixed Modeling (LCMM). Three distinct trajectories of CD40L expression on CD4^+^ T lymphocytes were identified: Class 1, Class 2, and Class 3. In the Class 1 trajectory, CD4^+^ T lymphocytes maintained consistently low CD40L expression levels. The Class 2 trajectory exhibited a U-shaped high expression pattern, with early high expression, followed by a marginal decline and a subsequent rise. The Class 3 trajectory presented consistently high CD40L expression levels (Figure 3A). To compare survival rates between different trajectories, Kaplan–Meier survival curves were plotted. The results displayed that patients in the Class 1 trajectory had significantly lower survival rates compared to those in the Class 2 and Class 3 trajectories (Figure 3B).

**Figure 3:**
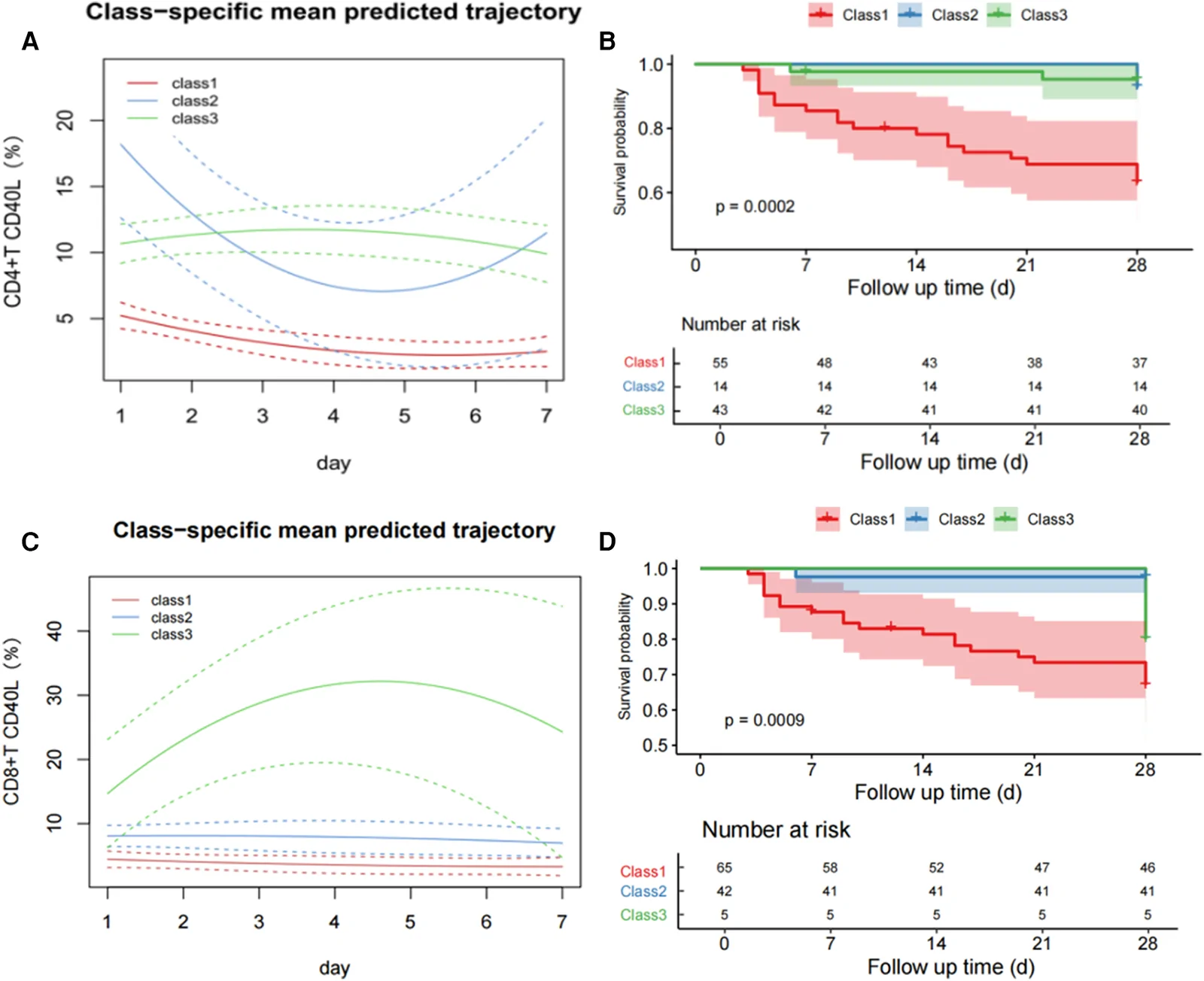
Trajectory patterns of T lymphocyte CD40L expression and Kaplan-Meier survival curves. A Class-specific mean predicted trajectory of CD4+ T cell CD40L expression. B Kaplan-Meier survival curves stratified by CD4 +T cell CD40L trajectory classes. C Class-specific mean predicted trajectory of CD8 +T cell CD40L expression. D Kaplan-Meier survival curves stratified by CD8 +T cell CD40L trajectory classes.

Similarly, three trajectories of CD40L expression on CD8^+^ T lymphocytes were identified in septic patients: Class 1, Class 2, and Class 3. In the Class 1 trajectory, CD8^+^ T lymphocytes maintained consistently low CD40L expression levels. The Class 2 trajectory showed relatively high expression levels, while the Class 3 trajectory exhibited an N-shaped high expression pattern, with early high expression, followed by a minimal increase (Figure 3C) and a subsequent decrease. Kaplan–Meier survival curves also delineated differences in survival rates among the three trajectories, with patients in the Class 1 trajectory having significantly lower survival rates compared to those in the Class 2 and Class 3 trajectories (Figure 3D).

### CD40L expression and 28-day mortality

Prior to exploring the relationship between CD40L and 28-day mortality in sepsis patients, machine learning algorithms were applied for feature selection to assess prognostic significance. In terms of feature selection, the Boruta algorithm, a widely used method for feature screening, was applied. The core principle of this algorithm is based on two concepts, namely “shadow features” and the “binomial distribution.” The Boruta algorithm generates a set of feature duplicates, referred to as shadow features, from the original dataset. If the Z-score of a given feature exceeds the maximum Z-score among the shadow features, it is considered important and retained; otherwise, it is excluded (12). However, the Boruta algorithm exclusively identifies important features without elucidating their specific impact. Therefore, to mitigate the inherent black-box nature of machine learning, SHAP (SHapley Additive exPlanations) was employed to calculate the marginal contribution of each feature value to the model's prediction, thereby aiding in interpreting the importance of each feature in the final prediction outcome and enhancing the interpretability of the model's results (13). Using the Boruta algorithm, eight variables significantly associated with 28-day mortality were identified: CD40L expression on CD4^+^ T lymphocytes on day 1, CD40L expression on CD8^+^ T lymphocytes on day 1, APACHE II score, SOFA score, mechanical ventilation (MV), use of vasopressors, aspartate aminotransferase (AST), and prothrombin time (PT) (Figure 4A). As depicted in Figure 4B, CD40L expression on CD8^+^ T lymphocytes had the most pronounced impact on 28-day mortality, followed by the SOFA score and CD40L expression on CD4+ T lymphocytes. In contrast, gender, APACHE II score, mechanical ventilation, and aspartate aminotransferase (AST) showed no significant effect on 28-day mortality.

**Figure 4:**
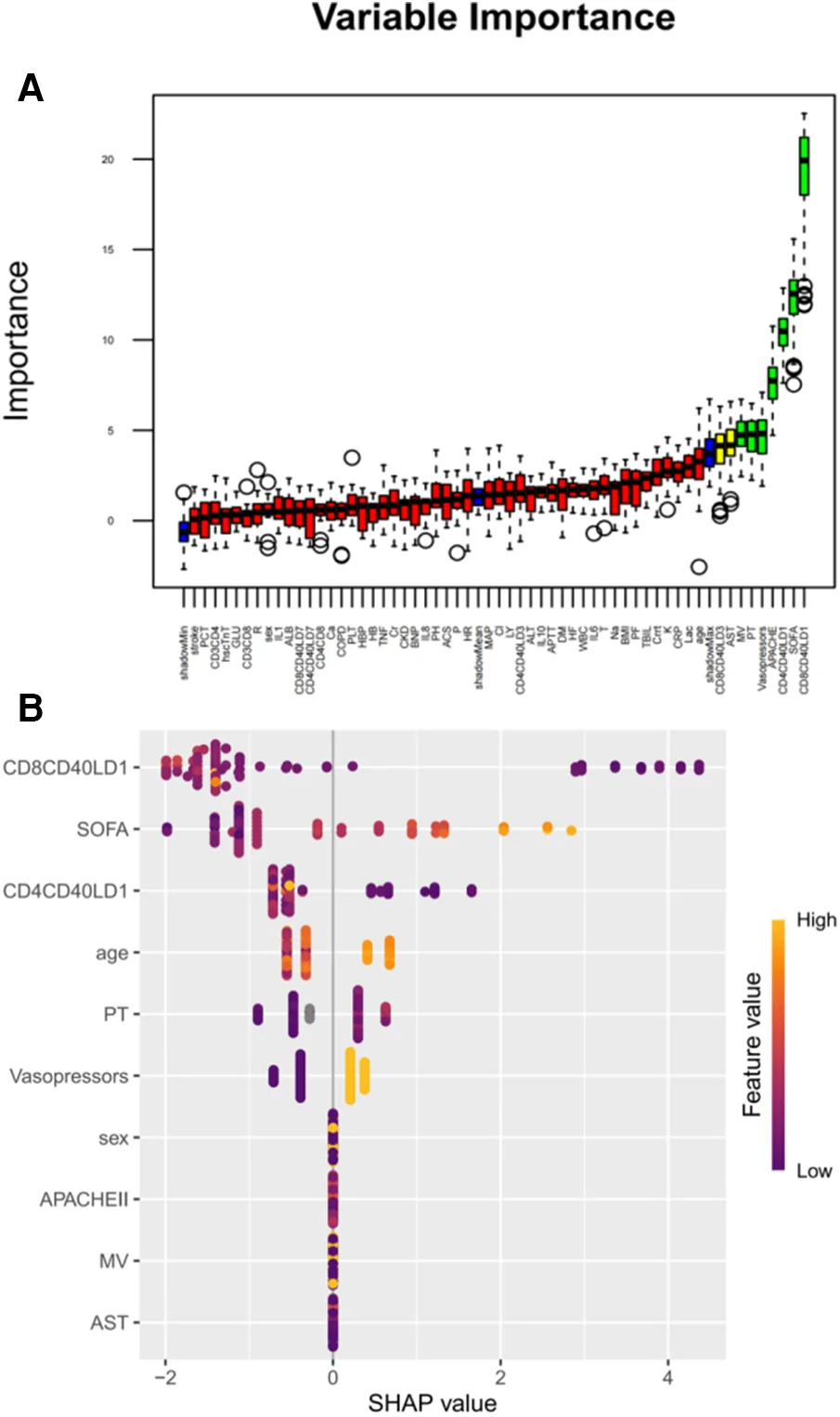
Application of machine learning in feature selection. **A** shows feature selection results using the Boruta algorithm to analyze the relationship between various clinical indicators and 28-day mortality. The *x*-axis denotes each variable, while the *y*-axis represents the Z-score of each variable. The box plots illustrate the Z-scores calculated by the model for each variable. Green boxes indicate important variables, yellow boxes indicate tentative attributes, and red boxes indicate unimportant variables. **B** presents the impact of each feature on the model output. Each point represents an individual patient, with the color indicating the feature value: yellow for higher values and purple for lower values.

Next, multivariable Cox regression analysis was conducted to investigate the association between CD40L expression levels and the risk of 28-day mortality in sepsis patients. In the present study, Model 1 included only the expression level of T lymphocyte CD40L, without any adjustment for other factors, to observe its unadjusted impact. In Model 2, two demographic variables, namely age and sex, were introduced as covariates. Model 3 was constructed based on clinical expertise and the results from the Boruta algorithm and SHAP analysis, incorporating several relevant variables, comprising age, sex, SOFA score, use of vasopressors, CD40L expression on CD4+ T lymphocytes on day 1, CD40L expression on CD8+ T lymphocytes on day 1, and prothrombin time. Multivariable Cox regression analysis revealed that CD40L expression levels on CD4 + and CD8+ T lymphocytes on day 1 were independently associated with 28-day mortality in sepsis patients. Higher expression levels were associated with a lower risk of mortality, indicating a protective effect. Specifically, the hazard ratio (HR) for CD40L expression on CD4+ T lymphocytes on day 1 and for CD8+ T lymphocytes on day 1 was 0.712 (95% CI: 0.606–0.837, *P* < 0.001) and 0.593 (95% CI: 0.481–0.731, *P* < 0.001), respectively. These findings were further validated in the fully adjusted model (Table 3).

**Table 3:** Association between CD40L expression and 28-day mortality.

Characteristic	Model 1		Model 2		Model 3	
	HR (95%CI)	*P*-value	HR (95%CI)	*P*-value	HR (95%CI)	*P*-value
28-day mortality						
CD4 ^+^ TCD40LD1	0.712 (0.606, 0.837)	<0.001	0.715 (0.609,0.840	<0.001	0.836 (0.714, 0.978)	0.025
CD8 ^+^ TCD40LD1	0.593 (0.481, 0.731)	<0.001	0.602 (0.488,0.741)	<0.001	0.719 (0.558, 0.927)	0.011

Model 1, unadjusted; Model 2, adjusted for age and gender; Model 3, adjusted for age, gender, SOFA score, use of vasopressors, CD40L expression on CD4+ T lymphocytes on day 1, CD40L expression on CD8+ T lymphocytes on day 1, and PT.

### Performance of CD40L

Thereafter, ROC curve analysis was performed to examine the predictive value of CD40L expression on CD4^+^ and CD8^+^ T lymphocytes on day 1, with SOFA and APACHE II scores used as reference indicators. The results indicated that the expression levels of CD40L on both CD4^+^ and CD8^+^ T lymphocytes on day 1 were closely associated with 28-day mortality in sepsis patients. The areas under the curve (AUC) were 0.847 (95% CI: 0.767–0.926) for CD4^+^ T lymphocytes and 0.875 (95% CI: 0.770–0.980) for CD8^+^ T lymphocytes. Meanwhile, the AUCs for SOFA and APACHE II scores were 0.864 (95% CI: 0.788–0.864) and 0.777 (95% CI: 0.675–0.879), respectively. More importantly, the AUC for CD8^+^ T lymphocyte CD40L on day 1 was higher than that for SOFA and APACHE II, while that for CD4^+^ T lymphocyte CD40L on day 1 fell between APACHE II and SOFA (Figure 5A).

**Figure 5:**
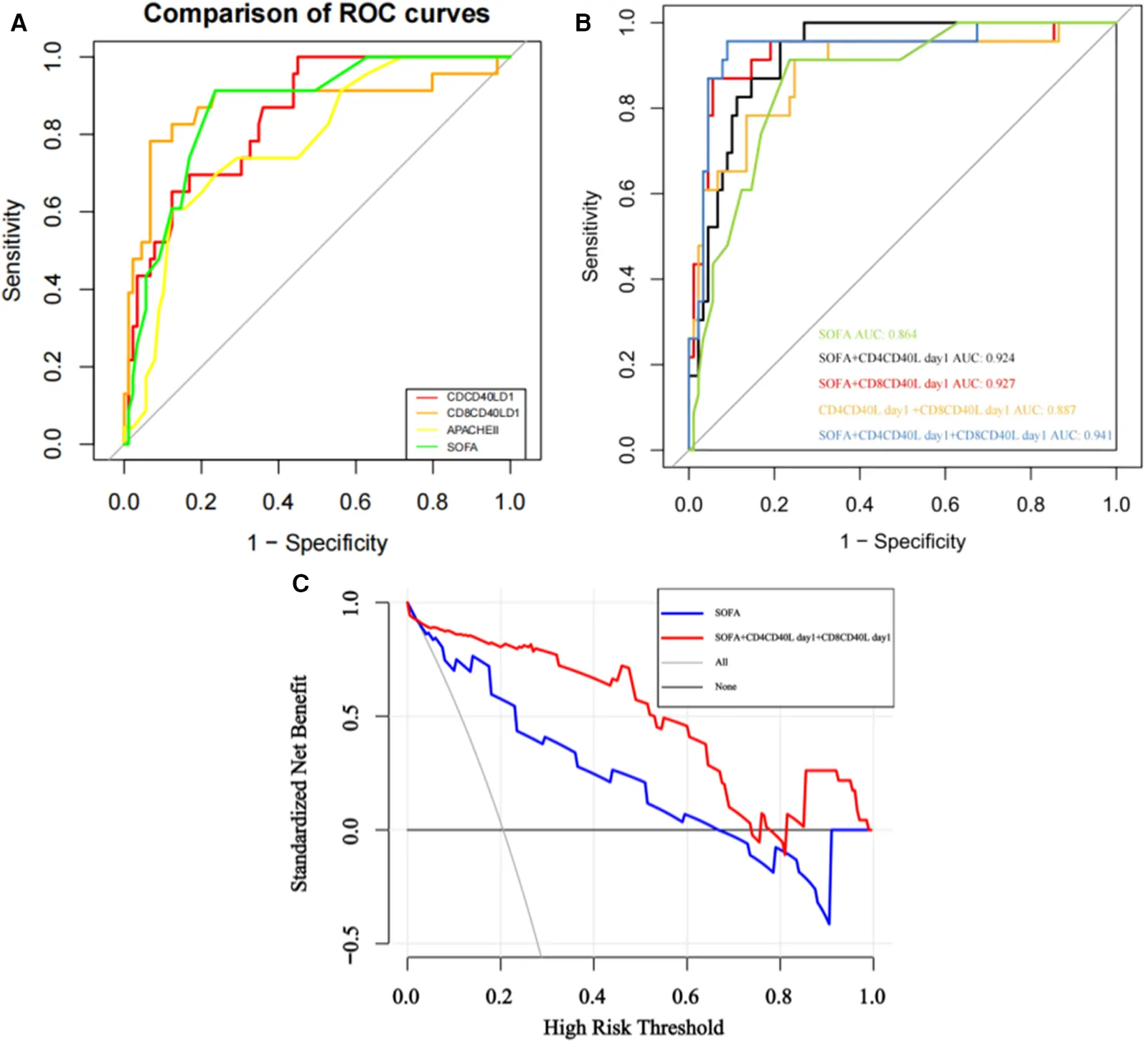
Performance of CD40L. **(A)** Area under the receiver operating characteristic (ROC) curve (AUROC) comparing the predictive accuracy of CD40L expression against SOFA and APACHE II scores; **(B)** ROC curve for the combined indicators; **(C)** Decision curve for CD40L expression on CD4^+^ T and CD8^+^ T lymphocytes combined with SOFA for predicting 28-day survival in sepsis patients.

Optimal cutoff values for predicting 28-day mortality in sepsis patients were as follows: 8.8% for CD40L expression on CD4^+^ T lymphocytes, 2.9% for CD40L expression on CD8^+^ T lymphocytes, 8.5 for SOFA, and 22.5 for APACHE II (Table 4). Therefore, CD40L expression on CD4^+^ and CD8^+^ T lymphocytes on day 1 was combined with the SOFA score to predict 28-day survival. As anticipated, the combined model yielded an AUC of 0.941, which outperformed the predictive power of any single indicator (Figure 5B).

**Table 4:** Comparison of the area under the curve (AUC) of CD40L on T lymphocytes and critical illness scores in patients with sepsis.

Indicator	AUC	Cut-off	95%CI	Specificity	Sensitivity	P
CD4 ^+^ TCD40Lday1	0.847	8.8	0.767–0.926	0.551	1	<0.001
CD8 ^+^ TCD40Lday1	0.875	2.9	0.770–0.980	0.933	0.783	<0.001
SOFA	0.864	8.5	0.788–0.864	0.764	0.913	<0.001
APACHE II	0.777	22.5	0.675–0.879	0.876	0.609	<0.01

The clinical utility of combining CD40L expression on CD4^+^ and CD8^+^ T lymphocytes on day 1 with SOFA was further assessed using decision curve analysis. The results demonstrated that the net benefit of the combined model was significantly greater than using the SOFA score alone, particularly in identifying high-risk patients (Figure 5C).

## Discussion

To the best of our knowledge, this is the first study to explore the relationship between CD40L expression on T lymphocytes and 28-day mortality in sepsis patients. Herein, the expression level of T lymphocyte CD40L was negatively correlated with the severity of sepsis and significantly associated with increased 28-day mortality. Notably, this association remained significant even after adjusting for various clinical and laboratory variables. Moreover, the dynamic expression trajectories of CD40L expression on T lymphocytes effectively distinguished different prognostic subgroups. These findings not only provide novel insights into the immunological mechanisms of sepsis but also offer potential targets for clinical prognostic assessment and individualized therapeutic interventions.

Herein, CD40L expression levels in peripheral blood lymphocytes were significantly elevated in sepsis patients, which is consistent with previous reports describing the role of CD40L in promoting inflammation and regulating immune responses in infectious diseases (11, 14–16). The activation of T lymphocytes is regulated by two distinct signals. The first signal is transmitted through the T cell receptor (TCR), while the second signal is provided by costimulatory molecules. As a key costimulatory molecule in T cell activation, CD40L binds to CD40 on the surface of antigen-presenting cells, promoting their activation and further enhancing the secretion of pro-inflammatory cytokines such as IL-6, IL-12, and TNF-α (17–20). According to a previous study, CD40L can be induced on dendritic cells by T lymphocytes, and even in the absence of CD4^+^ T lymphocytes, CD40L expressed on these dendritic cells can assist CD8^+^ T lymphocytes during the priming phase (21). A significant upregulation of CD40L expression was observed in the early stage of sepsis (within 24 h), suggesting its possible involvement in the initial phase of the cytokine storm. This temporal pattern is consistent with the findings of Michels et al. (2015) in a murine sepsis model, which demonstrated that inhibition of the CD40/CD40L axis alleviated encephalitis, oxidative damage, and blood-brain barrier dysfunction, as well as improved survival outcomes (11). This finding holds considerable clinical implications. First, T lymphocyte CD40L may serve as a biomarker reflecting real-time immune activation, potentially compensating for the limited specificity of traditional inflammatory markers. Secondly, the early peak in CD40L expression suggests that it could serve as a potential target for early immunomodulatory intervention in sepsis. In recent years, the CD40/CD40L pathway has emerged as a significant target in the treatment of autoimmune diseases, offering new perspectives for targeted immunoregulatory therapy in sepsis (15, 22).

Noteworthily, analysis of the dynamic changes in CD40L expression on days 1, 3, and 7 in patients with sepsis identified three distinct trajectories in both CD4 + and CD8+ T lymphocytes, highlighting the dynamic nature of immune regulation during sepsis. For CD4^+^ T lymphocyte CD40L, the patterns were persistently low expression and a U-shaped high expression pattern, characterized by high expression levels in the early phase, followed by a marginal decrease and a subsequent increase, and persistently high expression. For CD8^+^ T lymphocyte CD40L, the trajectories included persistently low expression levels, an N-shaped high expression pattern, hallmarked by high expression levels during the early phase, followed by a mild increase and a subsequent decline, and persistently high expression. Among these, the persistently low expression phenotypes of both CD4^+^ and CD8^+^ T lymphocyte CD40L were associated with 28-day mortality rates of 32% and 29%, respectively. Further analysis using Kaplan–Meier survival curves revealed that patients with the persistently low expression phenotype had significantly lower survival rates compared to the other two phenotypes. Of note, the persistently low expression of CD40L may reflect immunosuppression or an inadequate immune response. This is consistent with the findings of previous studies investigating sepsis-induced immunosuppression (SII), which is characterized by the release of anti-inflammatory cytokines, abnormal apoptosis of immune effector cells, excessive proliferation of immunosuppressive cells, and upregulation of immune checkpoint molecules (23, 24). Inflammation-related immunosuppression is a key factor contributing to secondary infections and multiple organ dysfunction syndrome (MODS). These patients are more susceptible to secondary infections or immune paralysis and have a higher risk of mortality (25).

Patients with high CD40L expression levels exhibited improved survival rates, which may be related to the following mechanisms: 1. CD40L assists in maintaining T cell function, promoting CD4+ T cell activation and cytokine secretion, and enhancing adaptive immune responses (21, 26, 27); 2. The CD40-CD40L axis plays a critical role in anti-infective immunity. CD40L interacts with dendritic cells and B cells, promoting antigen presentation and antibody production, which aids in controlling infection (28, 29); 3. CD40L governs endothelial function, the coagulation cascade, and platelet activation, thereby influencing the risk of microcirculatory dysfunction and organ failure in sepsis patients (30–32).

Importantly, the prognostic significance of CD40L may depend not only on its expression level but also on its temporal dynamics during the course of sepsis. CD40L plays an important role in T-cell activation and adaptive immune responses, but the CD40–CD40L pathway also has pro-inflammatory effects and may contribute to inflammatory signaling under conditions of excessive or dysregulated activation. Therefore, an early increase in CD40L expression may reflect active T-cell-mediated immune activation during the initial phase of sepsis, while excessive activation of this pathway may potentially contribute to hyperinflammation in certain clinical contexts. In our cohort, however, trajectories characterized by relatively high early CD40L expression, including the U-shaped trajectory of CD4+ T cells and the N-shaped trajectory of CD8+ T cells, were not associated with poorer outcomes compared with the persistently low trajectory. These findings therefore do not support a simple interpretation that early CD40L elevation is necessarily detrimental because of its potential pro-inflammatory effects.

Instead, the relatively high early expression observed in these trajectories may reflect preserved T-cell activation and adaptive immune responsiveness during the initial phase of sepsis. The subsequent decline and recovery observed in the CD4+ U-shaped trajectory, as well as the dynamic changes observed in the CD8+ N-shaped trajectory, may represent temporal adaptation of the host immune response, potentially involving fluctuations between immune activation and suppression. In contrast, persistently low CD40L expression may indicate sustained impairment of T-cell activation or evolving immunosuppression. Thus, the distinct trajectories identified in our study suggest that the prognostic significance of CD40L may be related to the dynamic pattern of its expression rather than to a single elevated or reduced measurement. Nevertheless, these mechanistic interpretations remain speculative, as our study did not directly assess T-cell functional capacity, detailed T-cell phenotypes, or downstream CD40–CD40L signaling. Therefore, the observed trajectories should be regarded primarily as prognostic patterns rather than direct evidence of distinct immunological mechanisms, and further longitudinal and mechanistic studies are warranted to clarify their biological significance.

In summary, exploring the dynamic expression of CD40L can reflect individual immune response patterns and provide a basis for precision stratified therapy. In this context, CD40L trajectories may have potential value for identifying distinct immunological phenotypes and informing future studies of individualized immunomodulatory strategies. However, whether patients with different CD40L trajectories would benefit from CD40L inhibition, immune stimulation, or other immunomodulatory approaches remains unknown and requires prospective mechanistic and interventional studies.

The findings of this study signaled that CD40L expression on peripheral blood T lymphocytes is significantly elevated in sepsis patients, with higher CD40L expression levels correlating with a higher survival rate. This finding suggests that during immune mobilization in sepsis, CD40L may enhance host defense against pathogens by activating immune cells, thereby improving patient prognosis.

However, a stronger immune response is not necessarily beneficial in all clinical contexts. Excessive or dysregulated CD40L signaling may potentially contribute to hyperinflammation and complications such as acute respiratory distress syndrome (ARDS) or acute kidney injury (AKI). This may be related to the bidirectional regulatory nature of the CD40/CD40L signaling pathway. Moderate activation may support coordinated innate and adaptive immune responses, whereas sustained overactivation may promote the release of inflammatory mediators, endothelial barrier disruption, and tissue injury. Therefore, maintaining a balance between appropriate immune activation and suppression is crucial. Further studies are needed to determine how CD40L-mediated immune activation can be appropriately regulated without causing excessive inflammation.

This study revealed the expression characteristics and clinical significance of T lymphocyte CD40L in patients with sepsis, contributing to individualized immune assessment. Additionally, the dynamic expression trajectories of CD40L can help identify high-risk patients and may offer immunomodulatory potential for those with poor prognoses. Nevertheless, several limitations of this study merit acknowledgment. First, as a single-center observational study, the results may be subject to selection bias and require validation in multicenter cohorts. Second, residual confounding by infection-related and treatment-related factors cannot be completely excluded. Although the multivariable Cox regression model incorporated seven clinically relevant variables, some potentially important factors, including infection site, pathogen type, and the appropriateness of empirical antibiotic therapy, were not included in the final model. Given the relatively small sample size, the inclusion of multiple covariates may also increase the risk of model instability and overfitting. Therefore, the association between CD40L expression and 28-day mortality should be interpreted with caution. Finally, although we characterized the dynamic trajectories of CD40L expression on CD4 + and CD8+ T lymphocytes, we did not further distinguish T-cell subsets or directly assess T-cell function and downstream CD40–CD40L signaling. Thus, the biological mechanisms underlying the distinct CD40L trajectories remain unclear. Future multicenter studies with larger cohorts and detailed T-cell phenotyping and functional analyses are warranted.

Future studies could focus on the following directions: developing rapid CD40L detection kits and validating their clinical utility; conducting interventional studies to evaluate the safety and efficacy of CD40L-targeted therapies; employing single-cell sequencing technology to analyze the transcriptomic characteristics of CD40L-high T cells; and exploring the co-regulatory network between CD40L and other immune checkpoint molecules.

## Conclusion

Peripheral blood T lymphocyte CD40L was identified as an independent predictor of 28-day mortality in **our single-center cohort** of patients with sepsis. Moreover, identifying distinct expression trajectories of CD40L in peripheral blood T lymphocytes can effectively predict short-term prognosis in patients with sepsis. Further multicenter studies with external validation are warranted to confirm the prognostic value and generalizability of these findings.

## Data Availability

The datasets presented in this article are not readily available because all raw clinical data involves patients’ private health information. Identifiable original data cannot be publicly published or uploaded to open repositories. Only anonymized summary data can be provided to researchers with legitimate ethical approval upon formal request to the corresponding author. The shared data is limited to non-commercial academic research and cannot be redistributed. Requests to access the datasets should be directed to Jun Wang, dr_wangjun@suda.edu.cn.
